# Supplementary material for: BITES: balanced individual treatment effect for survival data
Source: Bioinformatics. 2022 Jun 27;38(Suppl 1):i60–7. doi: 10.1093/bioinformatics/btac221 (PMC9235492; doi:10.1093/bioinformatics/btac221)
Supplement: btac221_Supplementary_Data [file btac221_supplementary_data.pdf]

Supplementary material for "BITES: Balanced Individual Treatment Effect for Survival data"

S1 Hyper-parameter tuning for simulation studies

To optimize hyper parameters in the three simulation studies, we used a comprehensive hyper-parameter grid search over the parameter values listed in Table S1. For each parameter combination, we fitted 50 initializations with randomized 60/40-train/validation splits. To avoid over-fitting we used early-stopping based on a non-improved validation loss over 50 consecutive epochs for all of the deep neural network recommendation systems ((T-)DeepSurv, SurvITE, (B)ITES). The best set of hyper-parameters was determined based on the minimal mean average C-index evaluated on the validation set. All presented results are based on an independent test set containing 1000 samples for each of the simulation studies, respectively. To reduce the computational burden of finding optimal IPM parameters (α and ϵ), we used the best set of hyper-parameters obtained by the corresponding model without IPM regularization ($\alpha = 0$).

Table S1: List of parameters used for the hyper-parameter grid search in the three simulation studies.

Hyper-parameters	Cox	RSF	T-DeepSurv<br>DeepSurv	SurvITE	ITES<br>BITES
Layers/Shared Layers	-	-	$\{[15, 10, 5], [10, 5]\}$	$\{[50, 50], [20, 20]\}$	$\{[15], [15, 10]\}$
Individual Layers	-	-	-	$\{[50, 50], [10, 10]\}$	$\{[10, 5], [5]\}$
Learning rate	$\{0.1\}$	-	$\{0.001\}$	$\{0.001\}$	$\{0.001\}$
Batch Size	-	-	$\{all\}$	$\{300, all\}$	$\{all\}$
l_2-Regularization	$\{0.1, 0.3, 0.5, 0.7\}$	-	$\{0.01, 0.1, 1\}$	$\{0.1, 0.01, 0.001\}$	$\{0.1, 0.01, 0.001\}$
l_1-Regularization	$\{0.01, 0.1, 1\}$	-	-	-	-
Dropout-rate	-	-	$\{0.1, 0.3\}$	$\{0.1\}$	$\{0.1, 0.3\}$
IPM strength α	-	-	-	$\{0, 0.01, 0.1, 1\}$	$\{0, 0.01, 0.1, 1\}$
Sinkhorn interpolation ϵ	-	-	-	-	$\{0.05, 0.1\}$
Number of Trees	-	$\{1000\}$	-	-	-
min samples split/leaf	-	$\{[6, 3], [12, 6]$<br>$[24, 12]\}$	-	-	-

For the SurvITE model training, we followed the example implementation[1]. Accordingly, we considered an over-parametrized architecture and scaled the outcome times to obtain 30 discrete time points. For IPM regularization ($\alpha \neq 0$), we used the predefined Wasserstein-distance.

S2 Small-sample-size simulation

The presented simulation studies in the main text show that in the non-linear and treatment-biased setting it requires at least ~ 1200 training samples to outperform the null hypothesis of always administering the treatment with the better average treatment effect (ATE). Here, we show corresponding simulation results for smaller samples sizes ranging from 120 to 480 training samples (Figure S2). For the linear simulation study, the treatment-specific Cox regression models show good performance in terms of C-index

[1] https://github.com/chl8856/survITE

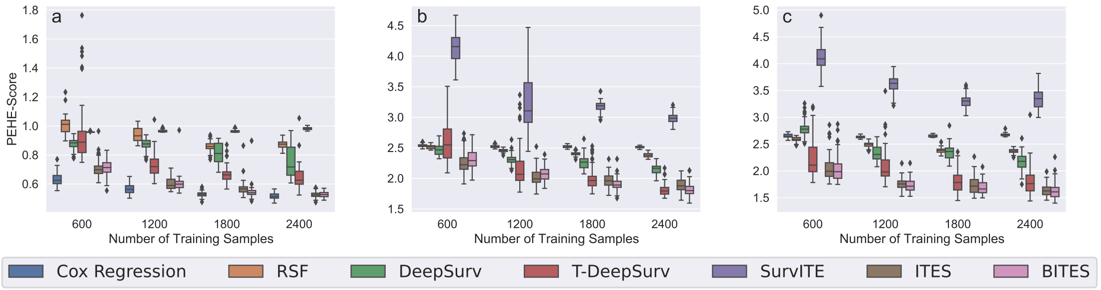

Figure S1: PEHE score obtained for the (a) linear, (b) non-linear and (c) non-linear treatment biased simulation. The boxplots give the distribution of PEHE-scores for 50 consecutive model initializations on independent test data using the best set of hyper-parameters.

for the whole range of training set sizes, which is also the case if we consider the proportion of correctly predicted "best treatments", which still outperforms the treatment recommendation based on the better ATE (the dashed horizontal line). However, we observed that for 360 and 480 training samples the Cox models are closely followed by BITES and ITES.

For the latter two simulation studies, i.e., the non-linear simulation study and the non-linear simulation study with treatment bias, none of the models, although they show reasonable performance in terms of C-indices, is able to outperform the null hypothesis of always administering the treatment with the better ATE (the dashed horizontal line). Therefore, we conclude that for the latter two simulation studies, none of the tested methods is able to provide proper treatment recommendations for small sample sizes between 120 to 480 training samples. Note, however, that this is highly dependent on the setup of the simulations studies. Here, multiple factors can affect results, such as the simulated effect sizes, the amount of censoring in the data, the effect size of the treatments (positive and negative) and the amount of non-linear covariate outcome dependencies.

S3 Supplementary information on "BITES optimizes hormone treatment in patients with breast cancer"

For the presented breast cancer application, we used data from the Rotterdam and the German Breast Cancer Study Group (GBSG), which are publicly available from the Comprehensive R Archive Network (CRAN)[2,3]. We performed the hyper-parameter grid-search for the Cox, RSF and SurvITE treatment recommendation systems over the parameter spaces shown in Table S3. For the remaining models, including (T)-DeepSurv and (B)ITES, we used the Asynchronous Successive Halving Algorithm (ASHA) imple-

[2] https://rdrr.io/cran/survival/man/rotterdam.html
[3] https://rdrr.io/cran/survival/man/gbsg.html

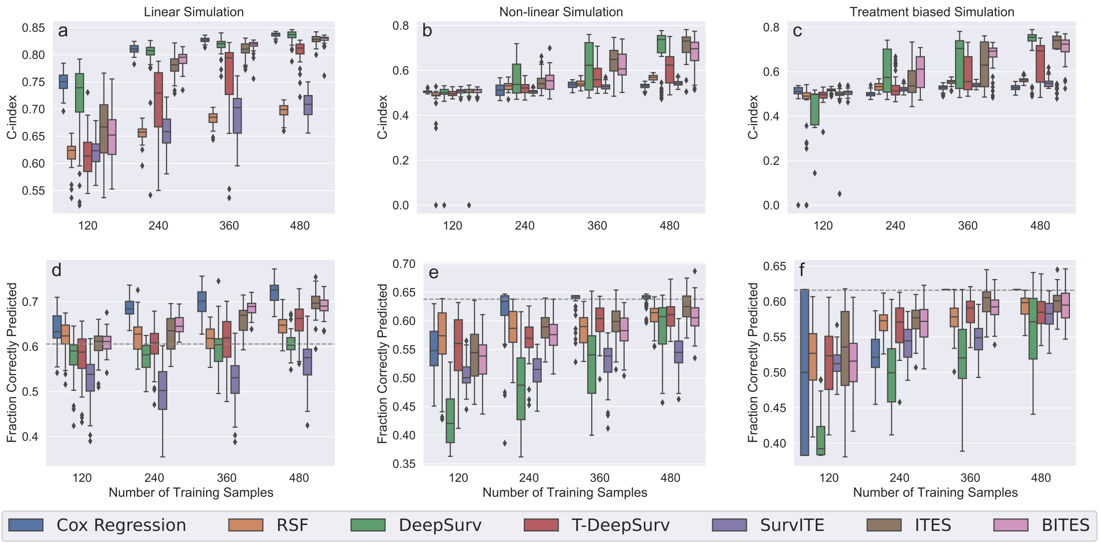

Figure S2: Harrell's C-index and the fraction of correctly predicted treatments for the linear (a,d), non-linear (b,e), and treatment biased non-linear (c,f) simulations with small training set sizes. The boxplots give the distribution for 50 consecutive simulation runs, i.e., for different model initializations, based on the best set of hyper-parameter determined by the validation C-index. Results are shown for different training sample sizes with 1000 fixed test samples for each of the simulations. The dashed horizontal line represents the fraction of patients that benefits from 100% treatment administration.

mented in the *ray[tune]* python package[4] for hyper-parameter screening, which lowers computation time by scheduled hyper-parameter optimization. There, we used a grid-search for structural parameters (i.e., network architecture) and the Sinkhorn parameters (i.e., α, ϵ), and allowed for random choices of the learning rate, l_2 regularizations and the dropout rate (the parameter search spaces are given in Table S2). For all models, we used 10 re-initializations with randomly drawn 80/20-train/validation splits. This yields 1236 training and 309 validation samples. Similar to the simulation studies, we avoided over-fitting by using early-stopping if the validation loss did not improve within 50 consecutive epochs epochs. The final models were selected by the minimal validation loss achieved for all of the evaluated hyper-parameter combinations and re-initializations. These models were then evaluated on an independent test cohort of 686 patients given by the GBSG Trial 2, with results shown in Figure 3, Figure S3 and Table 1.

[4]`https://docs.ray.io/en/latest/tune/index.html`

Table S2: Hyper-parameter search spaces for the hormone treatment optimization in breast cancer based on training data from the Rotterdam Tumour Bank.

Hyper-parameters	Cox	RSF	T-DeepSurv	SurvITE	(B)ITES
Layers/Shared Layers	-	-	$\{[7, 5]\}$	$\{[50, 50]\}$	$\{[7, 5]\}$
Individual Layers	-	-	-	$\{[50, 50], [10, 10]\}$	$\{[[5, 3], [3]]\}$
Learning rate	$\{0.1\}$	-	$[0.0001, 0.1]$	$\{0.001\}$	$[0.0001, 0.1]$
Batch Size	-	-	$\{all\}$	$\{300\}$	$\{all\}$
l_2-Regularization	$\{0.3, 0.5, 0.7, 0.9\}$	-	$[0.01, 0.1]$	$\{0.0, 0.01, 0.1, 0.5\}$	$[0.01, 0.1]$
l_1-Regularization	$\{0.1, 0.5, 1\}$	-	-	-	-
Dropout-rate	-	-	$[0.1, 0.2]$	$\{0.1\}$	$[0.1, 0.2]$
IPM strength α	-	-	-	$\{0.001, 0.1, 1, 10\}$	$\{0.001, 0.01, 0.1, 1, 10\}$
Sinkhorn interpolation ϵ	-	-	-	-	$\{0.05, 0.1\}$
Number of Trees	-	$\{100\}$	-	-	-
min samples split/leaf	-	$\{[6, 3], [12, 6]$ $[24, 12]\}$	-	-	-

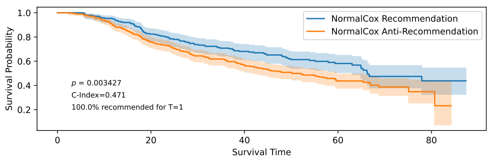

(a) Cox regression.

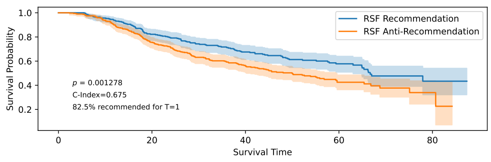

(b) RSF.

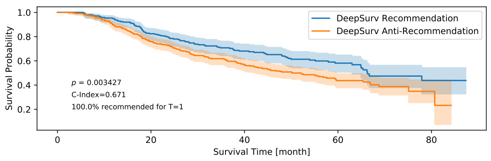

(c) DeepSurv.

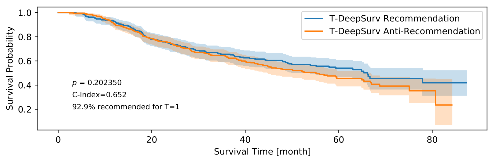

(d) T-DeepSurv.

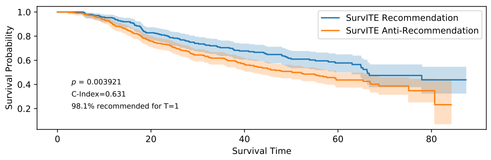

(e) SurvITE.

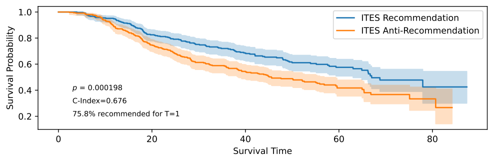

(f) ITES.

Figure S3: Kaplan Meier curves corresponding to Figure 3, for (a) Cox regression, (b) RSF, (c) DeepSurv, (d) T-DeepSurv, (e) SurvITE and (f) ITES. Each of the plots contains the p-value comparing the recommended and anti-recommended group, the obtained C-index and the fraction of patients that the algorithm recommends to administer the treatment.
